# Promotive and risk factors for children’s mental health—Finnish municipal policymakers’ and leading officeholders’ views

**DOI:** 10.1093/heapro/daac111

**Published:** 2023-03-01

**Authors:** Outi Savolainen, Marjorita Sormunen, Hannele Turunen

**Affiliations:** Department of Nursing Science, University of Eastern Finland, PO Box 1627, 70211 Kuopio, Finland; Institute of Public Health and Clinical Nutrition, University of Eastern Finland, PO Box 1627, 70211 Kuopio, Finland; Department of Nursing Science, University of Eastern Finland, PO Box 1627, 70211 Kuopio, Finland; Kuopio University Hospital, PO Box 100, 70029 Kuopio, Finland

**Keywords:** children, mental health promotion, municipal administration, socioecological model

## Abstract

Findings on children’s mental health promotion at the policy level are scarce, and the perceptions of the municipal administration on factors affecting children’s mental health have not been reported. This study describes the perspectives of policymakers and leading officeholders on promotive and risk factors for children’s mental health in a socioecological context. The perspectives of Finnish policymakers (*n* = 15) and officeholders (*n* = 10) in municipalities were examined using semi-structured interviews. The data were analyzed using inductive content analysis and were categorized according to the five levels of a socioecological model of health promotion: public policy, community, organizational, interpersonal and individual levels. The public policy level emerged strongly in the findings, specifically strategic planning and implementation challenges related to the promotion of children’s mental health in the municipality and state administration. At the community level, environmental factors promoting children’s mental health as well as risk factors were described. The organizational level consisted of support, requirements and development needs in children’s services. The importance of family and close networks at the interpersonal level, as well as the individual basis of mental health, were also evident. The integration and better collaboration of child and family services, the use of child rights impact assessment in political decision-making, and financial support from the state could contribute to improving strategic planning to support children’s mental health at the municipal level.

## INTRODUCTION

The basis of mental health (MH) is formed during childhood and adolescence. At best, the childhood environment supports MH and provides an opportunity for the positive development of mental resources (Vorma *et al.*, 2020). The goals of MH promotion are to strengthen mental well-being by creating supportive living conditions and environments and to reduce factors that are harmful to MH (Klemera *et al.*, 2017; Min *et al.*, 2017). In addition to genetic factors (Taylor *et al.*, 2017), there are a multiplicity of risk factors that can predispose children to negative outcomes—for example, pre- and peri-natal factors, such as young parental age (Oerlemans *et al.*, 2016) and maternal postnatal depression (Netsi *et al.*, 2018); individual factors, such as female gender (Merikukka *et al.*, 2018); family- and parenting-related factors, such as parental alcohol abuse (O’Hara *et al.*, 2019; Raitasalo *et al.*, 2019; Ukkola *et al.*, 2020) and environmental factors, such as seasonal changes (Wiens *et al.*, 2016). However, there are also promotive factors that can buffer these risks, such as healthy lifestyles (Bertoni, 2015; Mikkelsen *et al.*, 2017; Tamana *et al.*, 2019), positive relationships (Auersperg *et al.*, 2019), pupil participation in health promotion measures in the school context (Griebler *et al.*, 2017) and proximity to nature (Wiens *et al.*, 2016).

In Finland, as in many other European countries [e.g. (Pereira *et al.*, 2022)], municipalities have the overall responsibility for promoting the MH of children. Ways to promote MH include the prevention of bullying and discrimination, reducing poverty in families with children, making the strengthening of MH part of the culture of early childhood education and care (ECEC) and schools, and making work life more family friendly. The municipality needs to ensure that the conditions for well-being are taken into account in the activities of all administrative sectors and to organize appropriate social and health services (Health Care Act of Finland, 1326/2010; Local Government Act of Finland, 410/2015).

The Finnish National Child Strategy (Parliamentary National Child Strategy Committee, 2021), published in February 2021, emphasizes services that promote MH and treat MH disorders. In addition, the realization of the rights of the child requires the reform of services used by children (United Nations, 1989) and emphasizes the roles and rights of the child in administration and services. These efforts include considering and caring for the best interests of the child and supporting parents in their child-rearing tasks, including child protection and childcare services. Moreover, professionals in different fields need appropriate education, training and professional assistance to be able to meet the needs of parents and families (Sormunen *et al.*, 2011; Noonan *et al.*, 2019). Collaboration between different sectors is further emphasized in the healing and rebuilding needed to address the harmful consequences of the current COVID-19 pandemic (Guido *et al.*, 2020; Thualagant *et al.*, 2022), which has profoundly affected the lives of children and young people by increasing MH problems (Ministry of Social Affairs and Health, 2021; Wennberg *et al.*, 2020). Thus, such actions are necessary to secure children’s rights.

Despite the growing body of literature related to the factors affecting children’s MH and MH promotion in different settings, such as homes, schools, workplaces and communities, throughout their lives (Sharma *et al.*, 2017), findings on factors affecting children’s MH and MH promotion at the municipal policy level are scarce (Jenkins *et al.*, 2020). In general, there is a gap in the knowledge regarding the effects of political decision-makers and leading officeholders in municipal administrations on the well-being of children. The socioecological model (SEM) of health promotion (Stokols, 1996) is useful in explaining this complex phenomenon. The model covers both risk and promotive factors at five levels of the socioecological environment (i.e. individual, interpersonal, organizational, community and public policy) and is therefore appropriate for examining public health challenges too complex to be adequately understood and addressed from a single level, such as the MH problems of children. Consequently, the aim of the current study is to describe the perceptions of political decision-makers and leading officeholders on promotive and risk factors for children’s MH in a socioecological context.

## MATERIALS AND METHODS

Finland is a Nordic welfare society characterized by a public sector that offers citizens welfare services and a social safety net. Like all Nordic countries, Finland is a parliamentary democracy, with legislation requiring a majority in parliament (The Nordic Council and the Nordic Council of Ministers, 2021). Municipal administration, in turn, is based on the self-government of municipalities. The political decision-making power of the municipality is exercised by the municipal council. In addition, the municipality is required to have a municipal board and an inspection board. The council may appoint several committees, such as urban environmental, culture and leisure, education, social services and health committees. The municipal board and committees implement the decisions made by the council (Local Government Act of Finland, 410/2015).

Municipalities have a dual management system, in which local authority is characterized by division into political and professional management. Political management consists of decision-makers elected to municipal councils, boards and committees. Professional management, in turn, consists of leading officeholders who act as professional representatives of the administration and participate extensively in the various stages of the decision-making process in their area of administration. The officeholder organization manages the operational activities of the municipality (Constitution of Finland, 731/1999; Local Government Act of Finland, 410/2015). In this paper, the term policymaker refers to political decision-makers from municipal decision-making bodies, the term officeholders refers to the directors and leaders of different service sectors, and the term municipal administration refers to a management system consisting of political and professional management.

Using qualitative research methodology, open-ended semi-structured individual interviews were conducted in three municipalities in Finland. Purposive sampling was used to identify potential participants from sectors of growth and learning, well-being promotion, environment and social and health services, who would provide focused information on children’s MH promotion from the perspective of municipal administration (Curtis *et al.*, 2000). Policymakers’ and leading officeholders’ contact information was obtained from the public websites of the municipalities and municipal administrative services. First, the prospective participants received an information letter describing the study by e-mail. Altogether, 20 policymakers and 12 officeholders were contacted in person to ask about their willingness to participate in the study. Subsequently, 15 policymakers and 10 leading officeholders were interviewed by the first author. In later interviews, there were high degrees of repetition of the answers, and the amount of new information decreased. In the last two interviews, the views of the participants did not yield any new information. Therefore, data saturation was achieved by a total of 25, interviews and no more participants were recruited.

The interview guide (Table 1) was developed based on previous literature and the SEM of health promotion (Stokols, 1996). A pilot interview confirmed the feasibility of the interview guide. The interview topics were provided to the participants 1 week before their interviews to familiarize them. The interviews, which were conducted in Finnish by telephone between September and November 2019, lasted between 21 and 58 min and were recorded by the interviewer and transcribed by a professional transcription company; omissions in speech, word repetitions, missed syllables and single pronouns were omitted. Ethical approval for the study was obtained from the ethics committee of the university (statement 5/2019, 17.4.2019). In addition, a research permit was obtained separately from each municipal administration, and written informed consent was obtained from all participants.

**Table 1:** Interview guide for political decision-makers and officeholders

Topic	Subtopic
Background information	Municipality (working/serving as a municipal politician)
	Profession
	Role (officeholder/policymaker)
	Year of birth
	Work experience/experience serving as a municipal politician (years)
Mental health and mental health promotion of children in general	Definition of mental health and mental health promotion
	The role of the policymaker/officeholder in promoting the mental health of children
Factors affecting the mental health of children at different levels of the socioecological environment	Individual characteristics, development, diseases, etc., of the child (individual level)
	The child’s immediate environment, family, friends, etc. (interpersonal level)
	Early childhood education and care, basic education and primary health care (organizational level)
	Family support, inclusion, justice, physical environment, leisure activities, etc. (community level)
	Local decision-making and resources, national mental health promotion structures (public policy level)
Mental health promotion of children at the municipality	Early intervention in children’s mental health symptoms
	Children’s mental health services

The data were analyzed using inductive content analysis (Elo and Kyngäs, 2008; Vaismoradi *et al.*, 2013). The transcribed texts were read several times to obtain an overall impression, and the data were reviewed for their content. Meaning units were sentences or phases (Graneheim and Lundman, 2004), and they were chosen in line with the purpose of the study. Meaning units derived were condensed and coded for the identified categories according to the five levels of the socioecological environment (i.e. individual, interpersonal, organizational, community and public policy). In the analysis, the similarities and differences between the meaning units were compared, and categories and subcategories were created based on the comparison. An initial reading of the transcripts and preliminary coding were conducted in Finnish by the first author. The final analysis was validated by all authors. There was an ongoing dialog among authors during the final analysis process. By using Finnish in the analysis, the authors sought to ensure that the voices of the participants were accurately understood and represented. The English translation process was conducted during the preparation of this paper, and all authors participated in it.

## RESULTS

### General information

Participants (*n* = 25) consisted of policymakers (*n* = 15) and officeholders (*n* = 10), including both males (*n* = 13) and females (*n* = 12), aged 32–75 years. Leading officeholdersʼ work experience in their current position was 7.6 years, and municipal politicians had 9.1 years of experience. Policymakers were from municipal councils, municipal governments and committees. Officeholders were from different service sectors as part of the city’s service organization, including social and health services, growth and learning, well-being promotion and environment.

Policymakers and officeholders described promotive and risk factors related to children’s MH at the public policy, community, organizational, interpersonal and individual levels of the socioecological environment. Five main categories emerged from the descriptions of participants: (i) strategic planning and implementation challenges related to the promotion of children’s MH in the municipality and state administration; (ii) the contradictory roles of the environmental, building, culture and sport sectors in children’s MH promotion; (iii) support, requirements and development needs in children’s services; (iv) the importance of family and close networks and (v) the individual basis of MH. The main categories with subcategories are presented in Tables 2 and 3.

**Table 2:** The views of political decision-makers and officeholders on the promotive and risk factors for children’s mental health at the public policy and community levels

Category	Subcategory	Code
Strategic planning and implementation challenges related to the promotion of children’s mental health in the municipality and state administration	The personal, political and practical roles of municipal leadership in promoting children’s mental health	Defining municipal health promotion lines based on the opinions and values of policymakers and the perspective of the political group
		Local management as the role of senior municipal officeholders in health promotion
	The municipality’s weak resources in organizing services for children, parents and families	Insufficient or underused financial resources for the provision of child mental health services and services for parents and families
		Scarce human resources in student welfare services, social care and other child services
	Consideration of children’s mental health promotion in the municipality’s plans and the challenges of practical implementation	Consideration of children’s mental health in municipal strategies, local programs and plans, and the success of practical implementation
		A holistic view of the child and the family through the integration and better collaboration of child and family services and the steering boards
	Defining lines for the promotion of children’s mental health in central government and the contradiction of practical measures for the well-being of families	Financial support for municipal services for families with children and young people, early support and positive discrimination
		Consideration of the promotion of children’s mental health in national documents
		Reforming and strengthening legislation on the promotion of children’s mental health
		Measures to increase the poverty of families with children, reduction of municipal subsidies, cuts in mental health services and restriction of the subjective right to day care
The contradictory roles of the environmental, building, culture and sport sectors in children’s mental health promotion	The connection between the size and structure of the municipality, the presence and construction of nature and the well-being of children	Safe growth environment and fewer urban problems in small rural municipalities
		Consideration of child-friendliness in the location of schools, day care centers and parks
		Fewer service provisions and longer distances to travel in small rural municipalities
	Children’s opportunities to participate in hobbies	Children’s independent and organized sports, cultural and nature activities provided by the municipality, the parish, the third sector and associations
		Excessive competition, expensive fees, long travel distances, limited leisure activities and participation of all children

**Table 3:** The views of political decision-makers and officeholders on the promotive and risk factors for children’s mental health at the organizational, interpersonal and individual levels

Category	Subcategory	Code
Support, requirements and development needs in children’s services	Actions to support children’s mental health in early childhood education and care	Responding to basic needs
		Enabling the development of social skills
		Suitable facilities
		Small group sizes
		Adequate and qualified staff
		Safe boundaries and circadian rhythms
		Collaboration with child health clinic, social care and parents/guardians
	Actions to support children’s mental health at school	Small group sizes
		Guided activities
		Promotion of inclusion and equality
		Nonbullying
		Prevention of exclusion
		Teacher training and supervision
		Collaboration between school, other parties working with children and parents
	Actions to support children’s mental health in social and health care	Mapping the child’s situation, identifying risk factors and supporting the well-being
		Supporting parenting andthe well-being of families
		Collaboration between social and health care services
		Well-functioning child mental health services
	Development needs in social and health care	Developing a decentralized service network and processes
		Reducing the fear of stigma
		Investing in early intervention
The importance of family and close networks	Different family and home conditions	Caring, supporting and safe growing conditions
		An intact home
		Challenging life situations of parents/guardians
	Positive interpersonal relationships and networks of the child and the family	Adequate social contacts
		Friendships
		Functioning close network of the child and family
	Negative interpersonal relationships and networks of the child and the family	Bullying
		Experiences of externality and being alone
		Lack of close networks
The individual basis of mental health	Premise for the mental health of the child	Consideration of basic needs and individuality
		Opportunity for a safe childhood
		Meaningful activities
		Good self-esteem and inclusion
		Treatment of physical illnesses
		Holistic well-being
	Mental health challenges faced by the child	Inequality and economic values
		Social media and gambling
		Lack of meaningful activities
		Excessive demands
		Disregard for individuality
		Genetic factors

### Promotive and risk factors at the public policy and community levels

Political decision-makers and officeholders reported various factors that were associated with strategic planning and implementation challenges related to the promotion of children’s MH in the municipality and state administration and referred to the public policy level (Table 2). Municipal administration played a personal, political and practical role in children’s MH promotion. This role varied depending on whether the participant was a policymaker or officeholder. Municipal decision-making was felt to be largely based on pursuing the interests of one’s own political group. Policymakers stated that opinions, values and political perspectives influenced the municipality’s MH promotion policies.

The [political] group is really important in that.—It is, of course, of great importance whether you get the things that are important to you first through your own team and to the person who is on the decision-making board. But yes, that intergroup cooperation is done between parties…council groups (Policymaker 4).

The role of officeholders in children’s MH promotion, in turn, was related to local management, including directing resources, maintenance, control and information from child and family service entities, and various collaboration groups. Officeholders described being involved in the various stages of the decision-making process as professional representatives of the administration.

Participants saw the financial and human resources of the municipalities as poor or underused from the perspective of the welfare state in organizing services for children, parents and families. This meant that although the statutory basic services were properly organized, the focus was on addressing problems as they arose. In addition, interviewees felt that models of teamwork and multidisciplinary work were lacking and that the problem was operating by sector. Additional resources were sought for the affairs of individual sectors; the service package was not considered as a whole.

Regarding human resources, the officeholders’ view was that person-years have been added to the provision of services for children and young people and that human resources are in place in the service structures.

Well, if we think about this health sector now, then a huge number of person-years have been added to child and youth mental health services (Officeholder 1).

The policymakers saw the situation differently. They described a shortage of school curators and psychologists in student welfare services and a shortage of family counseling staff in social work.

Society’s resources are limited. Unfortunately, not all symptomatic children can be picked up. Yes, it would require more curators, psychologists and so on. Increasing the number would help (Policymaker 3).

Municipal strategies, programs and plans were largely described as good and taking children into account, but structures and resources were not always seen to match plans, and the system was fragmented and siloed. The integration and collaboration of child and family services was one way to improve the implementation of the plans through a holistic view of the child and the family that these services offer.

Participants described the important role of the central government in promoting children’s MH, both positively and negatively. Financial support for municipal services for families with children and young people, early support, and positive discrimination as an example were forms of support received from the state. In turn, measures taken by the state that negatively affected the well-being of families and thus for the MH of children were measures that increased the poverty of families with children, the reduction of municipal subsidies, cuts in MH services and restriction of the subjective right to day care.

In recent years, pretty bad decisions have been made nationally. This brings to mind, of course, livelihood-related decisions. This is, of course, the speech of a politician, but let me say that decisions to increase child poverty have been made in recent years, and they certainly have had bad effects (Policymaker 7).

Generally, participants were not satisfied with the promotion of children’s MH at the national level. Both national and international documents have addressed child well-being, but the existence of these documents was perceived as a separate matter from the actual guidance.

After all, there are a lot of these international documents and national documents that address the issue of children. Various articles and others. But I think that their existence is a little different than their real impact. How much do they really guide action, and are they really standards, or are they recommendations, and are they monitored? (Officeholder 3).

According to participants, activities were governed by laws and regulations, but legislation requires reform and tightening. Local decision-making values services and allocates resources. With such guidance, the promotion of children’s MH was not seen to be sufficiently effective in municipalities.

*The contradictory role of the environmental, building, culture, and sport sectors in children’s MH promotion* included factors related to the community level. Several participants referred to the size and structure of the municipality. The growth environment in small rural municipalities was perceived to be safer and closer to nature and to have fewer urban problems, such as crime and drug use. However, there were fewer leisure services, and there were fewer social and health care services in these municipalities, while the distances to these services were longer.

Participants described that child-friendliness should also be considered in environmental planning. This meant, for example, a stimulating daycare center environment. However, child-friendliness was not seen to be realized in the best possible way. For example, there were few places for youth to go. In addition, municipalities, parishes, third sectors and associations organized different hobbies for children. In some municipalities, leisure activities were limited, but there were other problems associated with the hobbies, such as fees that were too expensive, excessive competition and long distances to travel to the services.

Yes, I think there are [hobby opportunities] for children.—And there is not a long distance to travel to engage in hobbies for those of us who live in a population center. It is a different thing for those who live in those side villages. I can’t even think of their problems, but of course it does [long distance affects their participation] (Policymaker 11).

### Promotive and risk factors at the organizational, interpersonal and individual levels

*Support, requirements, and development needs in children’s services* refer to factors at the organizational level (Table 3). According to political decision-makers and officeholders, early intervention in children’s MH symptoms requires small group sizes and sufficient trained staff in the ECEC. Although participants emphasized the basics, such as dining, outdoor activities and a regular daily rhythm, and suitable facilities, such as spacious enough buildings, the importance of collaboration among sectors working with children, as well as collaboration with parents/guardians, also became clear.

Actions to support children’s MH at school were described somewhat similarly to those in ECEC. Multi-professional collaborations and collaborations between home and school were even more emphasized than in ECEC.

I think the key issue in the organization of basic education is inter-professionalism, as well as home–school communication, and learning and teaching the norms of the school community and, through it, the order of life. So, collaborative efforts have a substantial effect. In children’s mental health, they really do play a big role, helping children identify social boundaries and have confidence in their school community (Policymaker 10).

In addition, the role of schools in the promotion of inclusion and equality, the prevention of exclusion and the prevention of bullying were perceived as crucial.

Participants described that actions to support children’s MH in social and health care included actions targeted toward children, such as mapping the child’s situation, identifying risk factors and supporting well-being. However, the child’s MH was also promoted through parental support. Social security in particular was perceived to work with the whole family to support the well-being of the children.

Child health clinics and their services are those that very much promote the child’s mental health through parental support. There are many types of parent groups and operating models as well. And then, of course, on the child protection side, various subsidies for families, such as family work or home care for families with children, support family work, and family rehabilitation (Officeholder 5).

Child MH services in primary health care and child psychiatry functioned well for the most part, well but the problem consisted of long queues and long geographical distances to services. Developmental needs in social and health care were discussed, which could also contribute to solving the problems outlined above in child MH services. A decentralized service network and processes would help to perceive the family situations as a whole and could facilitate access to services.

But the fact is that this current approach is so fragmented that mental health promotion is not an intact whole. The child and family get small support crumbs here and there, but no one is watching the whole thing at the moment (Officeholder 9).

The development of services was seen as having the potential to reduce the fear of stigma associated with MH services and to contribute to the success of early intervention in cases where symptoms are observed in the child.

At the interpersonal level, *the importance of family and close networks* was emphasized. In the case of children in particular, different family and home conditions have a large role. Caring, supporting and safe growing conditions and an intact home were felt to support the child’s development. Challenging life situations of parents/guardians, substance abuse, MH problems and disadvantages, in addition to poor parenting skills of parents/guardians, were the largest risk factors at the interpersonal level.

Participants were also concerned about the intergenerational transmission of problems. Parents need support in challenging life situations, but they do not always have their own networks, such as close relatives. In this case, the child’s social networks were also narrowed, which could have contributed to the emergence of experiences of externalities. Strengthening friendships was considered important because being left alone, in addition to bullying, was felt to have a great impact on children’s MH.

It is really important that children are allowed to be with other children.—I think this kind of…it should be emphasized that children need—to make friends, or to allow them to be in contact with each other on a daily basis (Policymaker 5).

The premise for the MH and MH challenges faced by the child reflected the *individual basis of MH*. Policymakers and officeholders emphasized caring for the basic needs of the child. In addition to practical issues, such as treatment of physical illnesses, good self-esteem and inclusion in peer groups were mentioned as factors supporting the child’s MH as well as the right to be a child.

The right to be a child would probably be one thing that promotes mental health, accepting the things that they [children] want to do and giving them freedom in it (Policymaker 6).

Participants also mentioned various MH challenges faced by children. Although MH symptoms are due to genetic factors in some children, most of the risks are related to broader contemporary challenges, such as social media, or excessive demands related to, for example, school success or personal appearance.

In general, perhaps children today have too many performance goals, and they have been set too young. We are so busy that the child does not have time to spend their actual childhood so well (Policymaker 3).

## DISCUSSION

Several important findings related to the views of municipal political decision-makers and officeholders on the factors affecting children’s MH in a socioecological context arise from this study. The key findings at the public policy level were related to strategic planning and implementation challenges in the municipality and state administration. Participants expressed concern that the promotion of children’s MH was considered in the strategies, programs and plans of the municipalities, but the plans may not have been realized in practice. This has also been confirmed in a previous study (Savolainen *et al.*, 2021). One of the main reasons for this lack of realization was probably that services for children, parents and families were perceived as both dysfunctional and inadequately resourced. Policymakers in particular saw human resources as inadequate. Trained, competent and well-meaning personnel improve the quality and continuity of services and support the rights and well-being of the child (Noonan *et al.*, 2019). At present, personnel targets in social and health services are not fully realized, personnel are overburdened and turnover is high, and reducing turnover would help the child establish safe adult contact (Parliamentary National Child Strategy Committee, 2021).

From the global perspective, the situation in Finland regarding resources in organizing services for children is reasonable. However, interviewees stated that resources are used to deal with problems instead of for preventive services; this situation indicates a need for development considering that previous studies have reported the effectiveness of MH promotion as well as prevention of childhood mental illness (Raval *et al.*, 2019; Weare and Nind, 2011). In addition, participants perceived that the integration of fragmented services would allow a more holistic view of the whole family. In Finland, attention has been given to intact service packages in recent child strategy work. The strategy considers that the child- and family-oriented nature of services, along with accessibility and low-threshold services, should be developed with the help of the family center model (Parliamentary National Child Strategy Committee, 2021). Inconsistently, the state has reduced municipal subsidies, despite the fact that municipalities need financial support for development work. Budget cuts from efforts to support well-being are short-sighted because the cost of prevention shifts to repairing the harm, as seen from the financial cuts made during the economic depression of the 1990s in Finland, which increased the need for children’s MH services (Ristikari *et al.*, 2016). Thus, the child’s right to the best possible health care and adequate social and health services must be secured when planning possible budget cuts (United Nations, 1989; Constitution of Finland, 731/1999).

The roles of policymakers and leading officeholders in the MH promotion of children followed the traditional division. Policymakers described their role as ideological decision-makers who act as representatives of residents, especially their own constituents. Officeholders, in turn, identify themselves as local managers, including professional representations of the administration. In this way, officeholders were widely involved in the various stages of the decision-making process. Consequently, the MH promotion of children in the municipalities was influenced by the success of this dual management system. However, the turnover of policymakers is affected by the municipal elections held every 4 years (Local Government Act of Finland, 410/2015). As a result, the support of political parties may also change, which would change the municipality’s strategy and priorities in terms of children’s well-being. As officeholders also have a great influence in directing and implementing the MH promotion-related activities of the municipality, it should be ensured that they develop effective and long-term policies that address children’s health needs. However, previous studies have found that children and families with children are poorly reflected in political discourse and goal setting (Benning *et al.*, 2020; Hiilamo *et al.*, 2021; Pereira *et al.*, 2022).

The participants descriptions of the roles of the environmental, building, culture and sport sectors in children’s MH promotion appeared to be contradictory. Participants highlighted the strengths of small rural municipalities, such as proximity to nature, which has been found to increase well-being (Wiens *et al.*, 2016). However, children in small municipalities may be in an unequal position due to the reduced access to service provision and the long travel distances to services. Social inequality sharpens differences between children’s starting points, which can have far-reaching consequences (Vorma *et al.*, 2020). Thus, a growth environment that supports the positive development of mental resources should be invested in, for example, by developing the services of small municipalities to equal those of larger municipalities and by investing in e-health services that make social and health services more accessible to all (Risling *et al.*, 2017; Wynn *et al.*, 2020).

Several times, participants raised the importance of multi-professional and sectoral collaboration when they described support, requirements and development needs in children’s services. However, there is a need to develop the necessary knowledge and skills to promote effective collaboration across sectors (Tamminen *et al.*, 2022). Previous studies have found various factors, such as organizations’ different practices, lack of time, employees’ working habits and data protection, that prevent the transmission of information, which may pose a challenge to collaboration (Anderson *et al.*, 2017; Hoffman *et al.*, 2016). In addition, problems have been identified in the past in connections between basic and special services and between MH and other social and health services (Greene *et al.*, 2016). Alongside collaboration between professionals, participants also raised the importance of collaboration with parents. Such collaboration contributes to supporting parents in their upbringing work, as they play such an important role in children’s well-being (Sormunen *et al.*, 2011). This is a priority, especially for parents who have, for example, health-related problems, because parental health problems can also increase children’s subsequent MH problems and high-risk health behavior (Remes *et al.*, 2019). In addition, participants were concerned that the problems in the families, such as social disadvantages, were inter-generationally inherited. In socially disadvantaged families, preventive social work and preventive forms of child protection are important means of preventing the intergenerational transfer of problems (Vauhkonen *et al.*, 2017). According to participants, the basis of MH is built individually. Municipalities should consider the individual needs of every child in addition to universal measures. The best outcomes in terms of promoting children’s MH are achieved by incorporating both universal and targeted approaches (Weare and Nind, 2011). This means that the promotion of children’s MH and the planning of related services should, in principle, be based on knowledge of how children’s well-being is structured and what kind of support families with children need from society (Pekkanen *et al.*, 2020).

The results of this study highlight negative issues. Although, when compared internationally, the general levels of well-being and population health have continuously improved in Finland, the distribution of health and well-being in the population is increasingly unequal (OECD, 2019). The focus should be more on children who are in a worse situation. Several risk and promotive factors affect the overall MH of children. Thus, MH promotion should include both decreasing risk factors and increasing the number of protective factors. Not every factor that affects well-being can be fixed, such as possible genetic factors (Taylor *et al.*, 2017), but much can be done, such as taking the well-being of children into account in the activities of all administrative sectors and organizing social and health services that support the health of children and families (Vorma *et al.*, 2020).

This study has some limitations that need to be acknowledged and addressed. First, the results combine the responses of two different groups with different roles in municipal decision-making—policymakers and officeholders. Considering municipal policymakers and officeholders separately could have deepened the analysis of the views of a single group. However, there was a desire to obtain an overall picture, as both groups are involved in the decision-making process and the combined perspectives better answer the research question. Second, it is possible that participants with a particular interest in the topic were selected for interviews. This may have influenced some of the participants’ answers. Despite this limitation, both supportive and risk factors emerged equally from the results.

The trustworthiness of the study results was assessed through credibility, dependability and transferability (Graneheim and Lundman, 2004). Previous research on the promotion of children’s MH from the perspective of municipal policymakers and leading officeholders is scarce. Thus, the data were appropriate to collect through interviews. Participants were from different service sectors and from municipalities with different populations. This diversity supported the credibility of the study. The authors had previous experience in conducting qualitative research, and they had an open dialog within the research team, which strengthened the research’s dependability. In this paper, every stage of the study is described accurately, including a distinct description of the context, selection and characteristics of participants, data collection and process of analysis. Additionally, appropriate quotations are presented to help with the transferability (Elo and Kyngäs, 2008).

## CONCLUSIONS

This study describes the perceptions of municipal political decision-makers and leading officeholders on promotive and risk factors for children’s MH in a socioecological context. In general, the results highlight the need to promote children’s MH in municipalities. Most children in Finland are doing well, but support structures should target at the most vulnerable children to avoid the polarization of society and to support their parents, families and the children themselves. Specific, areas for development include the integration and better collaboration of child and family services and steering boards and the use of child rights impact assessments in political decision-making. Development requires action from the state, including legislative reform in addition to financial support.
